# Nano-scaffold-based delivery systems of antimicrobial agents in the treatment of osteomyelitis ; a narrative review

**DOI:** 10.1016/j.heliyon.2024.e38392

**Published:** 2024-09-24

**Authors:** Mina Yekani, Solmaz Maleki Dizaj, Simin Sharifi, Hossein Sedaghat, Mahmood Saffari, Mohammad Yousef Memar

**Affiliations:** a Department of Microbiology, Faculty of Medicine, Kashan University of Medical Sciences, Kashan, Iran; b Student Research Committee, Kashan University of Medical Sciences, Kashan, Iran; c Dental and Periodontal Research Center, Tabriz University of Medical Sciences, Tabriz, Iran; d Infectious and Tropical Diseases Research Center, Tabriz University of Medical Sciences, Tabriz, Iran

**Keywords:** Antibiotics, Drug delivery, Osteomyelitis, Nano-scaffolds

## Abstract

Osteomyelitis caused by drug-resistant pathogens is one of the most important medical challenges due to high rates of mortality and morbidity, and limited therapeutical options. The application of novel nano-scaffolds loaded with antibiotics has widely been studied and extensively evaluated for *in vitro* and *in vivo* inhibition of pathogens, regenerating damaged bone tissue, and increasing bone cell proliferation. The treatment of bone infections using the local osteogenic scaffolds loaded with antimicrobial agents may efficiently overcome the problems of the systemic use of antimicrobial agents and provide a controlled release and sufficient local levels of antibiotics in the infected sites. The present study reviewed various nano-scaffolds delivery systems of antimicrobial drugs evaluated to treat osteomyelitis. Nano-scaffolds offer promising approaches because they simulate natural tissue regeneration in terms of their mechanical, structural, and sometimes chemical properties. The potential of several nano-scaffolds prepared by natural polymers such as silk, collagen, gelatin, fibrinogen, chitosan, cellulose, hyaluronic, alginate, and synthetic compounds such as polylactic acid, polyglycolic acid, poly (lactic acid-co-glycolic acid), poly-ɛ-caprolactone have been studied for usage as drug delivery systems of antimicrobial agents to treat osteomyelitis. In addition to incorporated antimicrobial agents and the content of scaffolds, the physical and chemical characteristics of the prepared delivery systems are a determining factor in their effectiveness in treating osteomyelitis.

## Highlights

• Nano-scaffolds combined with antibiotics has widely been studied and extensively used for *in vitro* and *in vivo.*
• The present study reviewed various available nano-scaffolds for the delivery of antimicrobial drugs for osteomyelitis.
• Several nano-scaffolds prepared using natura and synthetic compounds as drug delivery systems for osteomyelitis.
• Incorporated antibiotics and the content of scaffolds are the determining factors in their therapeutic effects.

## 1 Introduction

Osteomyelitis is an inflammatory lesion in bone tissue that may be associated with increased rates of complicated bone injury, pathologic fractures, and sepsis. Osteomyelitis commonly progresses due to several mechanisms, including spread from blood to bone, spreading from neighboring tissue (due to a lesion or through from soft tissue), or a secondary infection following vascular diseases or neuropathy (such as diabetic foot infections (DFIs)). The bone infections may be local damage or may affect vast areas, including the bone marrow, cortical and trabecular bone, the periosteum, and neighboring tissues [[1], [2], [3]]. Numerous bacteria are involved in developing osteomyelitis, including Staphylococcus spp., Streptococcus spp., *Enterobacteriaceae,* and *Pseudomonas aeruginosa* [4,5]. Precise and timely identification of bone infections has been demonstrated to be problematic in healthcare centers [6]. Treatments for osteomyelitis are challenging, and nearly 40 % of the cases have the risk of recurrence and chronic infection. It has shown that an applicable treatment procedure is surgical debridement, especially for chronic cases, and antimicrobial drugs. Chronic osteomyelitis is problematic to eradicate, and it can considerably increase the financial burden. The frequency of chronic bone infections is growing because of the increased frequency of traffic accidents, orthopedic implants-associated infections, and DFIs. This is a significant challenge for physicians, principally when osteomyelitis is caused by drug-resistant microorganisms, especially methicillin-resistant *Staphylococcus aureus* (MRSA) [7].

The current treatments have some limitations, which may be associated with a vast lesion in bone tissue. It is difficult to attain a therapeutic level of antibiotics when administrated intravenously because systemic administration of antimicrobial agents can be associated with systemic toxicity. Blood flow restriction in bone tissue, emerging drug-resistant and biofilm-forming pathogens, and intracellular forms of pathogens are the most important factors that decrease the effect of antimicrobial agents and enhance osteomyelitis recurrence [5]. The most common antimicrobial agents that are used to treat osteomyelitis include vancomycin [8], clindamycin [2], rifampicin [9], linezolid [10], tigecycline [11], metronidazole and ciprofloxacin [12,13]. In the infected site, some molecules released by pathogens in the bone tissue trigger microbial biofilm formation, and the low pH of the injured site triggers this procedure together with stimulating the osteoclast activity and decreasing osteoblast activity, delaying bone healing [14]. The recurring nature, high morbidity, the need for prolonged hospitalization, and financial issues, have increased researchers' tendency to study novel technology to improve the methods involved in osteomyelitis treatment [15]. Treating chronic osteomyelitis using the local delivery of antibiotics is an innovative therapeutic approach, which realizes increased antibiotic levels at the infection site without systemic side effects [16]. The perfect local drug delivery systems for this purpose should be biocompatible, biodegradable, nontoxic, and effective in loading the broad-spectrum antimicrobial agents. To date, different antibiotics-loaded composite scaffolds have been studied to permit the controlled release of drugs for treating osteomyelitis. The scaffold is a 3D consistent and porous structure providing the appropriate surface for initial cellular attachment and growth, proliferation, and differentiation to form new tissue [17,18]. Even though several nanomaterials have been used to bone tissue engineering, their characteristics should be modified to mimic the tissue environments to induce fast interaction with cells surface and induce differentiate of precursor MSCs to accelerate osteogenic promotion [18]. Local drug delivery can overcome the microbial resistance challenge and decrease the toxicity of systemic administrated antibiotics. The treatment of bone infections using the local antimicrobial delivery approaches combined with the osteogenic scaffolds may efficiently overcome the problems of the systemic use of antimicrobial agents and provide a controlled release and sufficient localized levels of the antibiotics in the infected sites. If the delivery system contains osteogenic and antimicrobial materials, they can apply the dual purpose of eradicating the microbial agents and increasing tissue regeneration following surgical debridement. This paper is provided an outlook around the potential effects of antibiotics-loaded composite nano-scaffolds to give a prospect for their application as drug delivery system for antibiotics in the treatment of osteomyelitis. At first, bone histology and microbiology of bone infections were overviewed. Then, the efficiency of nanoscaffolds-based drug delivery systems for antibiotics according to their effects on microbial pathogens and bone tissue were discussed according to the previous reports. Therefore, data on nanoscaffolds-based drug delivery systems of antimicrobial agents for osteomyelitis were detected in databases of Google Scholar, PubMed, and Scopus. The searches were performed to detected published manuscripts with the keyword's osteomyelitis, nanoscaffolds, antibiotics and drug delivery. All English language articles were selected and checked independently by two authors.

## 2 Bone structure

**Fig. 1:**
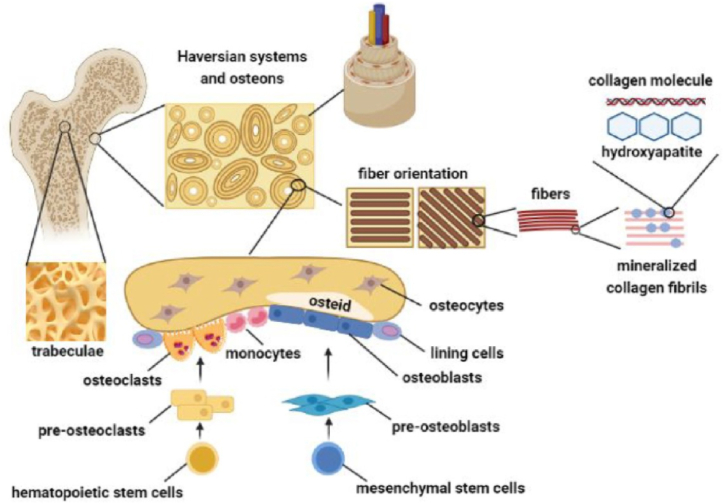
Bone histology and cells. Adopted from Ref. [19] with permission.

Bone is a viscous-elastic and mineralized connective hard tissue with an internal honeycomb shape matrix providing the rigidity of bone. Complex interactions between bone cells, matrix, and bone-derived and circulatory mediators are essential in bone construction and biological function. Bone cells are classified according to their locations, biological roles, and morphology into; osteoprogenitor cells, osteoblasts, osteocytes, and osteoclasts. Osteoprogenitor cells (mesenchymal stem cells) are precursors of osteoblasts. Osteocytes are derived from osteoblasts. Osteoblasts and osteocytes play a primary role in the bone tissue formation and mineralization. Osteoclasts are large multinucleate cells derived from blood-borne monocytes and involved in bone tissue resorption. The extracellular matrix between osteocytes space comprises sugars and polysaccharides, collagenous proteins (mainly type I collagen), special proteins, and leucine-rich proteoglycans on which calcium salts are deposited. The bone matrix is essential for the mechanical properties of bone, homeostasis, and remodeling process. It interacts with the osteocytes through adhesion molecules and affects bone cell activity (Fig. 1) [19,20].

## 3 Microbiology of bone infection

Both microbial factors and host conditions can play a determining role in developing osteomyelitis. Several bacteria are isolated from different types of osteomyelitis. However, *S. aureus* is the most commonly contributed in all types. Table-1 shows the common microbial pathogens and current antimicrobial agents for treating osteomyelitis. The bacteria express a range of virulence factors contributing to their pathogenicity in developing bone infections. Attachment to the extracellular matrix of host bone tissue and cells is the primary and essential step of the infection process, which is mediated by bacterial adhesions. After colonization, bacteria express different enzymes and toxins that are involved in tissue damage. Biofilm formation and metabolically altered forms (e.g., small-colony variants (SCVs) of *S. aureus*) contribute to the survival of bacteria and are associated with the persistence of bone infections. Biofilms protect bacteria from the damaging effects of antimicrobial agents and host immunologic response. Biofilms resistance to antimicrobial agents is revealed to be provided by several factors including decreased metabolic activity and low rates of cell division of deeply embedded cells in biofilm, and adaptive response to stressful conditions. Osteomyelitis is associated with an acute or chronic inflammatory response in which pathogens are embedded. Several inflammatory mediators, and the host immune cells themselves, contribute to tissue necrosis and damaging bone tissue. Bone necrosis may also develop due to ischemia because of compressed and obliterated vascular channels by the inflammatory response. Antimicrobial agents and inflammatory mediators cannot influence this avascular space, so treatment of osteomyelitis fails [1].

**Table-1:** Microbial pathogens and current antimicrobial agents for the treatment of different types of osteomyelitis.

Microorganisms	Osteomyelitis types	Drug of choice	Alternative
MSSA	Prosthetic-joint infection, Vertebral osteomyelitis, post-traumatic infection	Nafcillin or Oxacillin: 1–2 g IV Cefazolin: 1–1.5 g IV	Ceftriaxone: 2 g IV, Vancomycin: 1 g IV
MRSA	Prosthetic-joint infection, Vertebral Osteomyelitis, Post-traumatic infection	Vancomycin: 1 g IV For patients allergic to vancomycin: Linezolid: 600 mg IV	Minocycline: 200 mg orally initially, then 100 mg Fluoroquinolone: (e.g., levofloxacin, 750 mg) IV plus rifampin: 600 mg IV Linezolid: 600 mg (PO/IV) (or linezolid plus rifampicin PO: 600–900 mg) or daptomycin: 6 mg/kg IV
Streptococcus species	Prosthetic-joint infection, Vertebral Osteomyelitis, Diabetic foot infection	Penicillin G: 2 to 4 million units IV	Ceftriaxone: 2 g IV Clindamycin: 600 mg IV
*Enterococcu*s spp	Diabetic foot infection	Ampicillin: 2 g IV Ampicillin + gentamicin: 1 mg/kg IV/IM	Vancomycin: 15 mg/kg IV Vancomycin + gentamicin: 1 mg/kg IV/IM
*Enterobacteriaceae*	Prosthetic-joint infection, Vertebral Osteomyelitis, Post-traumatic Infection, Diabetic foot infection	Ticarcillin/clavulanate: 3.1 g IV Piperacillin/tazobactam: 3.375 g IV	Ceftriaxone: 2 g IV
*Pseudomonas aeruginosa*	Prosthetic-joint infection, Vertebral Osteomyelitis, Diabetic foot infection	Cefepime: 2 g IV plus ciprofloxacin: 400 mg IV Piperacillin/tazobactam: 3.375 g IV, plus ciprofloxacin: 400 mg IV	Imipenem/cilastatin: 1 g IV, plus aminoglycoside
Anaerobes	Post-traumatic Infection, Diabetic foot infection	Clindamycin: 600 mg IV Ticarcillin/clavulanate: 3.1 g IV	Cefotetan: 2 g IV Metronidazole: 500 mg IV
Mixed infection possibly involving (Anaerobe and facultative bacteria)	Prosthetic-joint infection, Vertebral Osteomyelitis, Diabetic foot infection	Ampicillin/sulbactam: 1.5–3 g IV Piperacillin/tazobactam: 3.375 g IV	Carbapenem antibiotic or a combination of fluoroquinolone plus clindamycin: 900 mg IV or metronidazole: 500 mg PO

IM-intramuscular, IV = intravenously.

## 4 Scaffolds in the bone tissue engineering

**Fig. 2:**
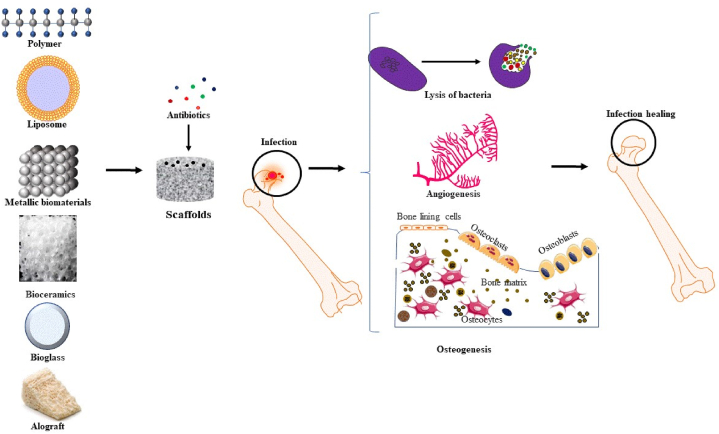
Application of scaffolds as drug delivery systems of antimicrobial agents for osteomyelitis treatment.

On an ongoing basis, a large number of surgeries are performed to replace tissue that has been damaged through infection or injury. Tissue engineering is a developing area in therapeutic sciences using materials and biological sciences to advance biological alternatives that reestablish, preserve, or recover cells and organs’ function. Practical tissue engineering needs suitable biomaterial scaffolds, appropriate cells, and cell growth inducers, along with progressive regenerative methods. Scaffold is a 3D matrix with a distinct macro, micro and, or nanostructure and an organized pore network that promotes the cells' attachment and growth of new tissue on its construct [[21], [22], [23]]. Scaffolds create a favorable environment for cell proliferation. They are commonly loaded with cells and occasionally cell growth inducer factors and drugs, or exposed to biophysical mediators in the form of a bioreactor or an approach, which uses various types of mechanical or biological stimuli to regenerate tissue [24,25]. Scaffolds can be used for bone tissue engineering if they achieve several criteria, including 1) a similar degradation level with the levels of bone tissue regeneration, 2) safety and biocompatibility, 3) having the capability of attachment to bone cells, and act as a surface for cell proliferation, 4) having interconnected porous construction with a mean diameter size of 100 μm or higher where more than half of space is naturally open to enable cell penetration and movement into the scaffolds [26]. [184]The[185] application of bone scaffolds combined with antibiotics has widely been studied and extensively used for *in vitro* and *in vivo* inhibition of microbial agents of osteomyelitis, regenerating the affected bone tissue and increasing bone cell proliferation (Fig. 2).

Biomaterials using technology of nanofabrication and nanoengineering offer promising biomimetic systems because they simulate natural biological tissues in terms of their mechanical, structural, and sometimes chemical properties. The adhesion and growth of cells with favorite capabilities can be controlled by the physicochemical properties of such 3D nano-scaffolds. Nanostructures with different shapes such as spheres, sheets, fibers, hollow tubes, and networks can be used to design scaffolds for orthopedic tissue regeneration such as bone, tendons, and cartilage [18]. Despite the remarkable advances in the design of nanoscaffolds in treating infections, these systems have limitations that must be resolved before their clinical application. Controlled release of antimicrobial agents to a specific region of the host body at a determined rate and within a certain duration is a limitation, and most antibiotics-delivery systems release antimicrobial agents regardless of time or host requirements [27]. Constant exposure to antimicrobial agents due to sustained release from nanoscaffolds can create a selective pressure, which plays a central role in the development of resistant strains [28]. In addition, a favorable antibacterial scaffold should not be toxic to host cells and not stimulate immune responses at a therapeutically used dose. The immunogenicity and toxicity of a delivery system may be affected by the presence of a therapeutic component and by the material formulation of carriers. For instance, Polyethylene glycol (PEG) is generally assumed as a non-toxic and well-tolerated compound that widely used in the formulation of scaffolds. However, [189] the induction of antibodies by PEG has been described, which can cause the clearance of a PEG compound and decrease the efficacy of the therapy [29].

### 4.1 Organic scaffolds

#### 4.1.1 Natural polymers

The potential of several natural polymers has been investigated in numerous biomedical applications, such as pharmaceuticals, tissue engineering scaffolds, drug carriers, and imaging. Natural polymers originated from different sources, such as plants, mammals, and microbes. Due to the high similarity of natural polymers with the extracellular matrix, bioactivity, high chemical and mechanical adaptability, safety, and high water-holding capacity, several studies have been performed for preparing scaffolds for tissue engineering purposes. Some natural polymer-based scaffolds with various structures have been described for usages in tissue engineering including solid porous, fibrous, hydrogel, and composites with decellularized materials (Table-2). Natural polymers generally are classified into two principal groups according to their origins including non-mammalian and mammalian-based polymers. Non-mammalian-based polymers include plants (starch), microorganisms (xanthan gum, gellan gum, and dextran), Marine algae (Carrageenans, Agarose, and Alginate), insects (silk fibrin), and crustacean (chitosan/chitin). The most common mammalian-based polymers are glycosaminoglycans (chondroitin sulfate, hyaluronan, and heparin) and proteins (collagen, fibrin, and elastin) [30,31]. The natural polymers can mimic numerous roles of extracellular matrix, so they improve cell adhesion and proliferation. However, natural polymers are not easy to engineer due to restricted processing facilities, high risk of their contamination, and a lot of variety [21].

**Table 2:** Application scaffolds designed by natural polymers in drug delivery of antimicrobial agents for osteomyelitis treatment.

Scaffold	Antibiotic	EE (%)	Release	Type of Study	Bacteria	Microbiological Tests	Animal Model	Treatment duration	Cell line	Proliferation	ALP	R
Silk fibroin	Vancomycin	94.2	50%–80 %/8 d^a^	*In vivo,* *In vitro*	MRSA	Agar diffusion, MIC and MBC	Rats	After 6 w	Rabbit osteoblast	No effect	Increased after 7 and 14 d	[40]
VEGF- loaded on silk fibroin	Vancomycin	97	20.38 %/24h, sustained/21 d	*In vitro*	MRSA	Agar diffusion	–	–	Human umbilical vein endothelial cells	Increased to >70 %	Increased after 7–28 d	[41]
Heparinized nanohydroxyapatite/collagen biocomposite	Vancomycin	–	Sustained/20 d^b^	*In vitro*	MRSA	MIC	–	–	Fibroblasts	–	–	[50]
Magnesium doped-hydroxyapatite collagen	Vancomycin, Gentamicin	–	Sustained 21d^b^	*In vitro*	*S. aureus, S. epidermidis* *E. faecalis, E. coli, K. pneumoniae, and P. aeruginosa*	Agar diffusion	–	–	MG63 Human Osteoblast-like Cell	Increased	–	[51]
Hydroxyapatite/Collagen Composites	Vancomycin	–	35 %/24h, sustained/120 h	*In vivo,* *In vitro*	MSSA	Agar diffusion, CFU count	Wistar rats	After 4 w	–	–	–	[52]
3D Printed Titanium Implants coated with collagen/hydroxyapatite	Vancomycin		0.07 ± 0.10 mg/L/7 d, no concentration 14 d	*In vivo*	*S. epidermidis*	CFU count, MIC	Rats	After 6 w	–	–	–	[53]
Porous gelatin-hydroxyapatite scaffolds CGHA1	Ciprofloxacin (1 wt%)	93.1	∼100 μg/mL/day	*In vitro*	MSSA, MRSA	Agar diffusion, CFU count, MIC	–	–	MSCs	No effect	Increased after 7 d	[58]
Porous gelatin-hydroxyapatite scaffolds CGHA2	Ciprofloxacin (2 wt%)	73.9	∼100 μg/mL/day									
Porous gelatin-hydroxyapatite scaffolds CGHA5	Ciprofloxacin (5 wt%)	58.4	150 μg/mL/day									
Porous gelatin-hydroxyapatite scaffolds CGHA10	Ciprofloxacin (10 wt%)	33.2	250 ± 50 μg/mL/day, Sustained/7 w									
Gelatin/genipin reinforced beta-tricalcium phosphate scaffold	Gentamicin	–	17 %/24h, sustained/28 d	*In vivo,* *In vitro*	MSSA, CoNS	Spread plate method, CFU count	Rats	After 3 w	–	–	–	[184]
Chitosan-calcium phosphate composite scaffolds (40/60 Cs to CaP)	Moxifloxacin	–	77.3/3d	*In vivo,* *In vitro*	MSSA, MRSA	–	Rabbits	After 4 w	MG-63 cell	Increased	Increased after 24 and 72 h	[77]
Chitosan-calcium phosphate composite scaffolds ((60/40 Cs to CaP))	Moxifloxacin	–	90.7 %/3d									
Chitosan scaffolds loaded with Zeolitic Imidazole Framework	Vancomycin	99.3	77 %/48h	*In vitro*	*S. aureus*	CFU count	–	–	MC3T3-E1	No effect	Increased after 14 d	[79]
Chitosan/nanohydroxyapatite/ethyl cellulose microspheres granules	Gentamicin	38.78	38 %/24h, sustained/49 d	*In vivo,* *In vitro*	*S. aureus*	CFU count, MIC	Rabbits	After 8 w	Rabbit osteoblast and human fibroblasts	–	–	[86]
Hyaluronic Acid Hydrogel	Vancomycin	–	65 %/24h, sustained/14 d	*In vivo*	MRSA	–	Sheep		–	–	–	[185]
	Gentamicin	–	74 %/24h, sustained/14 d									
Calcium-Alginate phosphorylated polyallylamine	Clindamycin	82.5	30 %/24h^c^	*In vivo* *vitro*	MRSA, *Enterobacter loacae*		–	–	MG63 cells	–	–	[110]

**Abbreviations:** ALP: alkaline phosphatase, CaP: calcium phosphate, CFU: colony forming unit, CGHA: Ciprofloxacin Loaded Gelatin–Hydroxyapatite, CoNS: Coagulase-negative staphylococci, EE: entrapment efficiency, MIC: minimum inhibitory concentration, MRSA: Methicillin-Resistant *Staphylococcus aureus*, MSSA: Methicillin-Sensitive *Staphylococcus aureus*, VEGF: vascular endothelial growth factor.

a pH 4.5 and7.4, respectively.

b The exactalues are not reported numerically in the text of referenced article and drug release patterns are presented in the graph.

c at pH:7

##### 4.1.1.1 Silk

Silk proteins are natural polymers from various spider and insect species, including fleas, ants, and crickets. For biomedical usage, silk is commonly prepared from the textile industry, such as silkworm *Bombyx mori* and sometimes spiders. Despite the higher strength and elasticity of silk sourced from spiders, silk obtained from *B. mori* is more available due to a significantly higher cultivating abundance. Fibroin and sericin are the two most frequent proteins of silkworm silk that belong to a glue-like family of proteins and comprise mainly Alanine, Glycine, and Serine. Fibroin and sericin are essential in coating the fibers and holding them together [32,33]. Several physicochemical properties and biological activities, such as mechanical hardness, biocompatibility, and safety, make silk fibroin proteins the suitable natural biomaterial for the slow and continued delivery of bioactive compounds and processed by different techniques to fabricate some drug delivery approaches such as microspheres, hydrogels, fibers, films and scaffolds [34,35]. However, resent *in vivo* study has shown low osteogenic properties and graft motion of silk fibroin textile prosthesis, resulting in joint injury/bone resorption and decreased mechanical fitness in animal model of anterior cruciate ligament [36]. For applications in bone tissue engineering, silk fibroin has commonly been used with other compounds with *in vitro* or *in vivo* beneficial effects on bone regeneration, such as calcium-phosphate and collagen [37]. Silk fibroin/chitosan composite nanofibers have been fabricated with the whole chitosan content of 0 %, 25 %, 50 %, 75 %, and 100 %, and the mean diameter sized from 215 to 478 nm that significantly induced *in vitro* proliferation and differentiation of Human fetal osteoblastic cells. Components of silk fibroin/chitosan composite nanofibers have shown different effects on the cell, with silk fibroin-triggered proliferation cell and chitosan-induced cell differentiation. Therefore, by selecting a desirable mix of these components in the composite, silk fibroin/chitosan composite nanofibers may be a promising biomaterial for bone tissue engineering [38]. The biphasic calcium phosphate/poly (ɛ-caprolactone) (BCP/PCL)–silk scaffolds have been fabricated with an appropriate internal construction characteristic of an interconnected porous network (99 % pore interconnectivity), and a total porosity of 85 % that significantly increased *in vitro* osteogenic gene expression and proliferation of human osteoblasts [39]. Silk fibroin nanoparticles have also been studied as a delivery approach for vancomycin with a >90 % EE and pH-dependent sustained release, favorable biocompatibility, and reduced infection in severe MRSA osteomyelitis in Male Wistar albino rats model relative to the untreated control [40]. Silk nano-scaffold has also been used for codelivery and a dual drug release system of vancomycin/and vascular endothelial growth factor (VEGF). VEGF has been loaded on silk fibroin nanoparticles (SFNPs) with particle size and zeta potential of 92 nm and-16.4 mV, respectively, and then embedded in vancomycin loaded silk scaffold that promoted the osteoblasts *in vitro* proliferation and inhibited infection [41].

Zhang et al. designed silk/nanosilver composite scaffolds containing gentamicin for chronic osteomyelitis caused by MRSA. AgNO3 was reduced with silk and formed silver nanoparticles that were uniformly distributed in the scaffold. Gentamicin was then loaded onto the scaffolds to treat osteomyelitis better. The *in vivo* examinations displayed the prevention of MRSA growth effectively [42].

##### 4.1.1.2 Collagen

Collagen is the most frequent protein in the animal kingdom that comprises about 20–30 % of total body proteins. Collagen plays a primary function in the mechanical function of the body tissues. More than 50 % of collagen in the body is found in the skin. Collagen is produced by fibroblasts, which commonly originate from pluripotential adventitial cells or reticulum cells [43]. Collagen fibrils and their networks are present in the extracellular matrix of body soft and hard tissues. Collagen is the most common component of bone together with hydroxyapatite (HAP) and constitutes about 90 % of organic materials and more than 30 % of volumetric composition of bone [24]. Thus far, 28 types of collagen have been recognized; types I, II, III, and V are the most common types of collagen in the bone and other mammalian tissues [44]. For biomedical usage of collagen, it is prepared from mammalian tissues such as the skin of porcine, decalcified bone or skin of bovine, bone of rabbit, and rat tail [45]. Collagen is also produced by recombinant technology, but it is not cost-effective, has a low yield, and is biologically inactive [46]. Because of desirable physicochemical properties such as considerable swelling capacity and significant biological activity such as low immunogenicity, impartibility, and regenerating ability, collagen is an extensively used polymer for different tissue engineering [47]. Some limitations of collagen have been addressed included poor mechanical stability, possible denaturation on processing, and the potential of inducing immune response and spreading infectious agents. Therefore, as an alternative option, instead of natural collagen obtained from animals’ tissue, recombinant collagen was suggested for tissue engineering procedures [48]. However, due to low mechanical strength, a pure form of collagen usually cannot be directly applied for bone tissue engineering. Therefore, it commonly is used to prepare composite scaffolds in combination with bioactive ceramics [49]. Heparinized nanoHAP/collagen composite has been fabricated in a granular form loaded with vancomycin for local delivery in the treatment of osteomyelitis and usage as a bone substitute. The nanoHAP/collagen delivery systems have released vancomycin with higher concentrations than concentrations need to inhibit both planktonic and sessile forms of MRSA, for 19 days. This system involved the local delivery of high levels of vancomycin to eradicate the infection and provided the regenerative activity to increase bone regeneration following antibiotic release [50]. Collagen fibers biomineralized with magnesium doped-HAP nanoparticles loaded by vancomycin and gentamicin have been prepared with a controlled release that provides a promising option for the inhibition of Gram-positive pathogens by vancomycin and Gram-negative pathogens by gentamicin during surgery without significant effects on biocompatibility and regenerative effects after antibiotics release. This study displayed that the MgHA/Coll hybrid scaffolds can be easily incorporated with antimicrobial agents, whose release is partly controlled through interaction with functional groups on the surface of MgHA particles [51].

The potential of HAP/collagen (collagen fibers with HAp nanocrystal deposits) has been studied for local drug delivery of different antimicrobial agents. After loading on HAP/collagen, minocycline, teicoplanin, and vancomycin have exhibited antimicrobial activity up to 2 weeks following subcutaneous implantation in animal models, while drugs with decreased absorbability included cefazolin, cefotiam, piperacillin has not shown a detectable antimicrobial activity. Vancomycin has been released from the HAP/collagen at levels higher than its MIC against *S. aureus* for seven days in the medullary space of the rat's femur, but cefazolin levels have not been detected. Parallel with these findings, embedding of vancomycin-loaded HAP/collagen in the rat femur with acute osteomyelitis has shown a higher significant therapeutic outcome relative to cefazolin, according to the CFU/mL counting of pathogens, the degree of tissue destruction, and tissue regeneration. These findings designated that the absorbability of antimicrobial agents onto their carrier is a critical factor when they are locally administered and that HAP/collagen nano-scaffolds might be a promising delivery approach for antibiotics in osteomyelitis treatment [52]. The 3D printed titanium implants coated with collagen/HAP nanofibers impregnated with 10 wt % of vancomycin have shown the ability of osteointegration and inhibit bone destruction in animal models infected by *S. epidermidis* [53].

##### 4.1.1.3 Gelatin

Gelatin is a protein originating from Type I collagen through partial hydrolysis using gradual melting. Gelatin has the advantage of being less expensive and uncomplicated than collagen and limitations similar to collagen included the poor mechanical parapatries and partial immunogenicity [48,54,55]. Gelatin has been extensively studied for use in 3D cell culture as a matrix and in tissue engineering as a constituent of scaffolds [56,57].

The scaffolds composed of porous gelatin–HAP have been prepared with several concentrations of ciprofloxacin (1, 2, 5, and 10 wt%). Released ciprofloxacin from these scaffolds has been reported to penetrate human adipose-derived mesenchymal stem cells, significantly decrease the amount of MRSA and Methicillin-susceptible *S. aureus* (MSSA), and increase the osteogenic differentiation ability of infected cells without adverse *in vitro* effects on normal human adipose-derived mesenchymal stem cells [58]. Scaffolds of gelatin-agarose-contented glass nanoparticles have been prepared as a delivery vehicle for ciprofloxacin. The use of glass has improved drug release profiles from the scaffolds and their ability to precipitate a HAP layer on their surfaces [59]. Mesoporous silica nanoparticles (MSNs) have been widely used in the biomedical field because of their desirable properties such as, tunable size and pore volume, high specific surface, easy functionalization, and excellent biocompatibility [60]. Surface-functionalization MSNs have been studied to loading and controlled the release of different bioactive molecules because of the morphology and pore size of MSNs. Multifunctional (magnetic and luminescent) MSNs have allowed simultaneous bio-imaging and drug delivery. The modification of MSNs, with suitable functional groups (such as–NH2, –Cl, –SH, or –CN) or polymers, significantly affects the cargo release pattern by increasing the drug circulation resistance [61]. The composite scaffold has been prepared based on gelatin matrix and vancomycin-loaded MSNs. The addition of MSNs with a size of 209.8 nm to gelatin has led to a significant increased pore size. An increased pore size from 90.7 ± 23.5 to 121.4 ± 35.1 μm has been reported by increasing MSNs content from 5 to 20 %, making the scaffolds more suitable for vascularization and tissue growth in bone tissue engineering. With the incorporation of MSNs, the vancomycin-MSNs/Gelatin composite scaffold showed an improved release pattern of vancomycin. It exhibited a significantly decreased primary release, finally revealing a long-term inhibitory effect on bacterial proliferation, as demonstrated by *in vitro* antibacterial effects on *S. aureus* [62].

##### 4.1.1.4 Fibrinogen

Fibrinogen is a pleiotropic and soluble plasma glycoprotein essential in blood clot formation, inflammation, and tissue repair [63]. Fibrinogen synthesis occurs primarily in hepatocytes [64]. Fibrinogen synthesis is considerably enhanced in response to inflammation [65]. Fibrinogen 3D scaffolds with immunomodulatory properties have been fabricated for bone regeneration/repair at six days (acute phase of inflammation) and eight weeks (repair of the bone defect) post-injury, together with the local and systemic immune responses [66]. Increased bone regeneration effect has been observed by fibrinogen on chitosan scaffold [67]. Because fibrinogen has various favorable features in bone repair/regeneration including slow drug release and increased cell proliferation, it has been broadly studied to use in biomaterial science for bone tissue engineering and orthopedic surgery applications [68]. Fibrinogen scaffolds have some disadvantage, such as poor mechanical strength and high degradation rate, which can be improved through mixing with other compounds and various cross-linking methods, respectively [69]. Fibrinogen is used to modify chitosan to increase induction of bone regeneration and mediator of angiogenesis *in vitro* than chitosan substrate [70]. Nano-scaffold composed of chitosan nanofiber/fibrinogen has been made with the ability of sustained release of platelet-derived growth factor (PDGF) for the induce of fibroblast and has been reported to be highly efficient as bioactive dressings for the increase of tissue healing [71].

##### 4.1.1.5 Chitosan

Chitosan is a linear polysaccharide containing randomly arranged β-linked D-glucosamine and N-acetyl-D-glucosamine, achieved by the alkaline deacetylation from chitin. Chitin is the most frequent component of marine crustacean shells, such as crabs, shrimps, lobsters, and prawns [34]. Chitosan is an extensively used polymer in tissue engineering biomaterial due to its favorable biocompatibility and high biodegradability, with promising properties such as safety, high drug loading capacity, the ability to adhesion to the mucous membrane, at least one of which is a mucosal surface and high charge density to apply in pharmaceutical usage [72]. Chitosan is an attractive natural material as a bone tissue biomaterial because it induces the adhesion and proliferation of osteoblast cells and supports the development of mineralized bone matrix [73]. It is renewable and shows an appropriate moisturizing and adsorption feature [74]. Some disadvantages of chitosan films are weak mechanical features, low solubility, a high degradability rate, low thermal stability, difficulty in its electrospinning and controlling pore size, and water resistance [75,76].

Moxifloxacin-loaded Chitosan-based calcium phosphate composites have boosted *in vitro* MG-63 osteoblasts proliferation/differentiation and prevented post-operative osteomyelitis caused by *S. aureus* in an animal model [77]. HAP-chitosan nanocarrier has been prepared for dual and controlled delivery of sodium alendronate and gentamicin that exhibited significant antimicrobial effects against *S. aureus* and *Escherichia coli* in a concentration-dependent manner. Co-delivery of antimicrobial agents and antiresorptive compounds using bioresorbable chitosan has increased the treatment efficiency of osteomyelitis due to inhibiting bacterial growth and inducing bone formation [78]. Chitosan scaffolds loaded with Zeolitic Imidazole Framework-8/vancomycin have been introduced that can be considered as a potential drug carrier and bone substitute to be utilized in the treatment of sever bone infections caused by *S. aureus* [79].

##### 4.1.1.6 Cellulose

Cellulose is the most frequent biomaterial on the Earth that can be prepared from a broad spectrum of sources such as cell walls of wood and plants, some prokaryotes, and algae, as well as tunicates, which are the only identified animal sources of cellulose [80,81]. Pure cellulose is not biodegradable in the human tissues, has a low osseointegration and triggers inflammatory response that restrict its clinical usage as scaffolds [82]. Cellulose derivatives are more attractive than pure cellulose because of their higher solubility in water and common organic solvents. Cellulose derivatives are an attractive option for the biomedicine applications and bioanalysis fields due to their considerable biocompatibility, biodegradability, safety, low immunogenicity, thermo-gelling behavior, mechanical properties, availability, cost-effectivity, biological effects, antimicrobial activity and high hydrophilicity [83,84]. Dual functional nano-scaffolds have been prepared from chitosan, carboxymethyl cellulose, and silver nanoparticles and provided improved mechanical properties and antimicrobial effects due to decorated silver nanoparticles on carboxylated, which treated bone tissue infections such as osteomyelitis caused by *E. coli* and *Enterococcus hirae and* increased *in vitro* growth and adhesion of MG63 cells [85]. Microspheres granules containing chitosan/nano HAP/ethyl cellulose impregnated with gentamicin with encapsulation amount of 38.78 ± 0.23 and release pattern of 38 % at the first day, shown significant therapeutic effects on a rabbit model with *S. aureus* chronic osteomyelitis without cytotoxic effects for fibroblast and osteoblast [86]. Vancomycin release system using hydroxy propyl methyl cellulose (HPMCs) and chitosan/glycerophosphate (Ch/Gp) thermosensitive hydrogel has been designed with a polymer/drug ratio of 1/1, spherical and smooth shape, particle size of 1.5–6.4 μm and loading capacity of 63 % for the local treatment of osteomyelitis. Vancomycin/HPMCs have shown initial burst and fast release 80 % and 92 % of drug release within eight and 12 h, respectively that may be attributed to the high hydrophilicity of HPMC microparticles. The release pattern of vancomycin from Ch/Gp has been significantly decreased (42 % and 94 % within eight and 72 h, respectively), indicating that the Ch/Gp hydrogel has a considerable effect on vancomycin release [87].

##### 4.1.1.7 Hyaluronic acid

Hyaluronic acid (HA) is a long, unbranched polysaccharide polymer of repeating disaccharides of D-glucuronic and N-acetyl-D-glucosamine that originate from the human tissues as well as be obtained through microbial fermentation [88,89]. HA plays an essential structural role in tissue due to specific and non-specific interactions with the extracellular matrix, crucial to cellular signaling transduction with particular molecules and receptors [90]. HA has some disadvantages included weak mechanical properties, hydrophilic nature, and fast degradation *in vivo,* which can improved by chemical modification or crosslinking [91]. CD44 is the primary cell surface receptor interacting with HA [92]. Interactions between CD44 and HA occur within the HA-binding domain (HABD) [93]. CD44 is known to play a vital role in the extracellular matrix synthesis and organization, and it plays also an important role required for some cellular procedures, such as morphogenesis, proliferation, and skin injury healing. CD44 is extensively expressed in several cells, such as mesenchymal stromal cells [94]. Hydrogels consisting of HA have been widely studied to develop tissue engineering biomaterials [95]. HA also has anti-adhesive and antibacterial effects, two noticeable advantages for tissue engineering biomaterials at injury sites. Because of its hydrophilic properties, antimicrobial effects of HA have been reported on common pathogens of bone infection, such as Staphylococcus, β-hemolytic *Streptococcus*, *P. aeruginosa*, and *Enterococcus* [96].

##### 4.1.1.8 Alginate

Alginates are high-swelling natural anionic polysaccharides, which are known to have favorable biodegradability and safety [97]. Alginate is naturally obtained from the cell wall of brown macroalgae (Phaeophyceae) and some red algae and capsules of bacteria, including *Azotobacter* spp. and *Pseudomonas* spp. [98,99]. The presence of carboxyl groups at alginate molecule, which is charged at pH higher than 3–4 and causes its water solubility at neutral and alkaline conditions, plays a vital role in the extensive application of alginates and makes it an appropriate option for drug delivery systems [100]. However, immunogenicity, the slow degradation rate and absence of cell attachment sites for osteoblast anchorage can be considered as the limitations of alginate-based scaffolds [101,102]. Alginates nano-based materials have been developed to encapsulate antimicrobial [103,104], anti-anaphylactic [105], inflammation inhibitory [106], reactive oxygen species (ROS) [107], and antioxidant compounds [108].

Dual-drug-based scaffold encapsulating polycaprolactone-based scaffold containing cephazolin and a rifampin-loaded alginate hydrogel have been fabricated and studied to inhibit biofilm and planktonic *S. aureus*. This scaffold exhibited prolonged antimicrobial properties by providing sustained release of cefazolin via the encapsulation of rifampicin. This scaffold has shown a synergistic antimicrobial effect due to the antibiofilm effect of rifampicin and the antibacterial effect of cefazolin [109].

Scaffolds composed of clindamycin-loaded nano calcium alginate phosphorylated polyallylamine have exhibited significant antimicrobial effects on MRSA with MIC of 275 μg/mL, and *E. cloacae* with MIC of 120 μg/mL, and exhibited inhibitory effects on the bacterial biofilm formation, motility, and exopolysaccharides formation. Clindamycin-loaded nano calcium-alginate phosphorylated polyallylamine has been reported to be biocompatible in several concentrations and shows 99 % cell viability of MG63 during the 24 h [110]. Sodium alginate has also been used with porous silica nanospheres to encapsulate green tea polyphenols. The outer layer of prepared polyphenols-loaded sodium/alginate/porous silica nanospheres microgel spheres have been further enveloped by pH-sensitive CaCO_3_ that is used to deactivate the acidic condition triggered by bacterial growth, and encapsulated TP has been released pH sensitively to tolerate oxidative stress. This system has exhibited appropriate drug delivery features, including drug loading efficiency of 92.96 % and drug loading content of 19.62 %. Sodium/alginate/porous silica nanospheres/CaCO_3_ has effectively inhibited *S. aureus* growth and induced the growth and differentiation of osteoblasts in exposure to H_2_O_2_ at a pH of 5.5 [111].

#### 4.1.2 Synthetic polymers

Synthetic polymers have been developed to use in biomedicine and gained noticeable attraction for medical usage for different reasons including an extensive variety of physical and chemical features that can be attained according to the monomer components, polymerization methods, and establishment of co-polymers containing diverse components at variable levels [112]. The most considerable advantage of synthetic polymers is their multipurpose performance. Some properties of polymers such as physicochemical characterizations, biocompatibility, and biodegradability are highly influenced by their molecular size and composition, which can be controlled according to certain requirements. However, the absence of cellular receptors and the inability of inducing cell signaling pathways and subsequently deficiency of cell response to the synthetic polymer are limitations of this type of material in the biological application. The degradation of synthetic polymers is driven mainly by hydrolysis, while natural polymers are degraded in enzymatic ways or combined with hydrolysis [22]. There are different kinds of synthetic biodegradable polymers, such as polylactic acid (PLA), polyglycolic acid (PGA), poly-ɛ-caprolactone (PCL), poly-b-hydroxybutyrate (PHB), and poly (lactic acid-co-glycolic acid) (PLGA) [113]. Some synthetic polymers have been widely used to fabricate scaffolds for drug delivery systems of antimicrobial agents for osteomyelitis treatment (Table 3).

**Table 3:** Application scaffolds designed by synthetic polymers in drug delivery of antimicrobial agents for osteomyelitis treatment.

Scaffold	Antibiotic	EE (%)	Release	Type of Study	Bacteria	Microbiological Tests	Animal Model	Treatment outcome	Cell line	Proliferation	ALP	R
Hydroxyapatite- polylactic acid	Vancomycin	–	–	*In vivo*, *In vitro*	*S. aureus*	–	Mice	After 2 w	Human osteoblasts	Increased	–	[186]
Polylactic acid/Titanium dioxide (TiO2)	Norfloxacin	30.75–40.77 ≠.	90 %/1080 h	*In vitro*	*S. aureus, P. aeruginosa, E. coli, Salmonella* and *K. pneumonia*	Agar diffusion	–	–	HepG, HCT, MCF-7, PC-3, Hela, WISH, WI-38	–	–	[124]
Hydroxyapatite/Poly (lactic-co-glycolic acid)	Gentamicin	42.43–49.85	75.0 %/48h, sustained/17 d	*In vitro*	*S. aureus*	Agar diffusion	–	–	MC3T3-E1	Increased at 14 d	–	[187]
Biodegradable poly-ε-caprolactone/poly (lactic-co-glycolic acid)	Tobramycin (50 mg/mL)	–	47.8 %/3 d, sustained/33 d	*In vivo,* *In vitro*	*S. aureus, E. coli*	Agar diffusion	Rats	After 8 w	–	–	–	[188]
	Tobramycin (25 mg/mL)	–	46.3 %/3 d, sustained/51 d									
Electrospun polycaprolactone (PCL)	Vancomycin	–	70 %/12h, Sustained/14 d	*In vivo,* *In vitro*	*S. aureus*	CFU count	Rabbits	12 w	Osteoblast	No effect	–	[146]

**Abbreviations:** ALP: alkaline phosphatase, CFU: colony forming unit, EE: entrapment efficiency.

##### 4.1.2.1 Polylactic acid (PLA)

PLA is a hydrolysable polyester consisting of lactic acid (2-hydroxypropionic acid) monomers prepared from renewable agricultural leftover due to the fermentation of the starch present in sugarcane and corn [114]. PLA is one of the most widespread polymers, producing 0.2 million tons in 2015 and 0.3 million tons in 2019 [115]. PLA is approved by the FDA (mostly known as safe), sustainable, biodegradable, and compostable compound with an appropriate mechanical feature. All these significant properties make PLA an attractive material for bone tissue engineering applications [116]. PLA biocompatibility [117], toxicity [118], physicochemical and mechanical properties are influenced by assortment of monomers, polymer size, copolymerization, and functionalization. PLA has been approved by FDA for human usage as joins and bone implants and in preparations for controlled delivery systems for various drugs and vaccine antigens [119]. Some disadvantages have been addressed for PLA included low degradation rate, low cell attachment, and insufficient osseointegration due to its hydrophobicity. PLA also induce the inflammation due to the acidic degradation products [120]. These restrictions limit PLA's usage in bone-regenerative approaches, especially if specific interactions between host cells and scaffolds are required [120,121]. The hydrophobicity decreases the PLA penetration in living cells [120]. Hydrophobic surfaces of PLA may cause bacterial adhesion and provides desirable environment for biofilms formation [122]. Currently, to overcome the disadvantages of PLA in the biomedical field, chemical processing, composite technology, and novel fabrication methods are being used to improve PLA effects. Nano-hydroxyapatite PLA with size of 100 nm loaded with vancomycin has been shown meaningfully higher infusion efficacy than PEI, empowering it to efficiently transport vancomycin to bone marrow, consequently improving antimicrobial effects and decreasing expression of CD4, CD8, CD19, and CD20 in the damaged site of MRSA chronic bone infection in the animal model. Nano-hydroxyapatite PLA significantly has preserved osteoblast viability and triggered its proliferation and migration thus improved repairing damaged bone tissue. Moreover, in animal model, Nano-hydroxyapatite PLA -vancomycin has been suppressed inflammatory response and enhanced the number and depth of bone trabeculae [123]. Norfloxacin-loaded PLA/TiO_2_ nanocomposites have considerable antimicrobial effects on *S. aureus, P. aeruginosa, E. coli, Salmonella* and, *K. pneumonia* [124].

##### 4.1.2.2 Polyglycolic acid (PGA)

PGA has a comparable construction to PLA without the methyl side chain, which permits the polymer chains to pack together closely and leads to more hydrophilic, elevated levels of crystallinity (45–55 %), high thermostability (Tm more than 200 °C), extraordinarily barrier properties to CO_2_ and O_2_ (more than EVOH), as well as high tensile strength (115 MPa) and stiffness (7 GPa) [125]. PGA is formed by the polycondensation procedure between glycol and aliphatic dicarboxylic acids. The constituents originated from renewable sources, such as glycol from glycerol, and organic acids obtained via fermentation [126]. Some disadvantages are addressed for PGA included difficulties in monitoring mechanical stability and the porous structure properties including pore connectivity, and pore size and spatial distribution [127]. PGA is commonly used as a filler compound incorporated with other degradable polymers. It is frequently integrated into biomaterial scaffolds for different tissue engineering usage. However, PGA has some limitations such as its quick degradation could potentially induce unfavorable inflammatory reactions related to the enhancement of glycolic acid. PGA has been combined with collagen to prepare a bone tissue engineering scaffold seeded with mesenchymal stem cells and studied in animal models of bone critical-size defects. The collagen/PGA scaffold and the mesenchymal stem cells-seeded collagen/PGA scaffold have significantly increased bone healing [128].

##### 4.1.2.3 Poly (lactic-co-glycolic acid) (PLGA)

Poly (lactic-co-glycolic acid) (PLGA) is a linear copolymer produced by ring-opening co-polymerization of two different proportions of its integral monomers, glycolic acid and lactic acid (LA) and glycolic acid (GA) [129]. Among numerous synthetic polymers, PLGA has gained considerable attention due to its satisfactory degradation features and biocompatibility. PLGA has widely been studied for its potential to encapsulate different bioactive molecules and drugs, such as antibiotics, DNA, cisplatin, docetaxel, and curcumin [130]. However, clinical usage of pure PLGA for bone tissue engineering is limited by the low osteoconductivity and undesirable mechanical features for usage as the load-bearing approach. Thus, it is commonly has been studied in incorporated with other compounds, or it is altered to increase PLGA biomimicry and bioactivity and improve its ability in bone regeneration [131]. PLGA microspheres have been used for loading silk fibroin-coated vancomycin with a drug loading of 24.11 ± 1.72 % and encapsulation efficiency of 48.21 ± 3.44 %. Silk fibroin has alleviated the initial vancomycin burst release in varying degrees [132].

Qiao et al. prepared a composite scaffold with anti-osteoporosis and anti-infection effects. The 3D printing method was used to prepare the PLLA/Pearl scaffold. To formulate the PLLA/Pearl/RM-P scaffolds, microspheres of rifampicin/moxifloxacin-PLGA (RM-P) were loaded into the pores of the scaffold. The PLLA/Pearl/RM-P and PLLA/Pearl scaffolds promoted the proliferation and differentiation of bone marrow mesenchymal stem cells. The scaffolds were implanted into the bone nidus of the rabbit model. PLLA/Pearl/RM-P scaffold showed bone defect repair and anti-infection effect [133].

In the last two decades, solid and liquid lipids have been widely used for many nanoparticulate formulations of nanospheres and nanocapsules [134]. Solid lipid nanoparticles (SLNs), previously referred to as lipospheres [135], are one of the promising systems to deliver bioactive compounds because of favorable biodegradability and safety [136,137]. Lipid liquid-crystalline nanoparticles have been studied to deliver teicoplanin for treating *S. aureus* caused chronic osteomyelitis [138] (Table 4). Several formulations of SLNs containing several lipids including Imwitor, Stearic acid, Softisan and Dynasan have been used to deliver ciprofloxacin using the ultrasonic melt-emulsification technique. Ciprofloxacin-loaded Stearic acid-containing SLNs have shown the optimum formulation, which exhibited a relatively low polydispersity index of 0.05, size of 174 ± 0.9 nm), zeta potential of −23.4 ± 0.02 mV, and highest nanoparticle yield (73.66 %) and entrapment efficiency of 73.94 %. Moreover, ciprofloxacin-loaded Stearic acid-containing SLNs have shown not only the ideal drug release pattern but also higher inhibitory effects on osteomyelitis caused by *P. aeruginosa* and *S. aureus* [139]. The hybrid nanoparticle of lipid-polymer-contained lipids-coating PLGA loaded by linezolid (LIN-LPN) has been prepared that showed significant *in vitro* higher inhibitory effects against intracellular and biofilm-forming MRSA than free linezolid. A low lipid: polymer ratio in LIN-LPN has been shown to be associated with smaller size, but the PDI increased significantly when this ratio was lower than 0.2 likely because of decreased stability [140].

**Table 4:** Application scaffolds designing by organic, inorganic and bioglass biomaterials drug delivery of antimicrobial agents for osteomyelitis treatment.

Scaffold	Antibiotic	EE (%)	Release	Type of Study	Bacteria	Microbiological Tests	Animal Model	Treatment outcome	Cell line	Proliferation	ALP	R
Solid lipid nanoparticles formula containing stearic acid (CIPSTE)	Ciprofloxacin	73.94 %	Sustained/12 h	*In Vitro*	*S. aureus, P. aeruginosa*	CFU count	–	–	–	–	–	[139]
Hybrid nanoparticle of lipid-polymer contained lipids coating PLGA	Linezolid	51.4 ± 1.33 %	30–40 %/12 h, sustained/5 d	*In vivo,* *In vitro*	MRSA*, S. epidermidis*	CFU count, MIC	Rats	–	MC3T3-E1osteoblasts	–	–	[140]
Mesoporous silica nanoparticles and gelatin matrix	Vancomycin	–	19 %/24h, Sustained/28 d	*In vivo,* *In vitro*	*S. aureus*	Agar diffusion	Rabbits	After 4 w	BMSCs	Increased	Increased>7 d	[62]
Mesoporous silica microspheres/nano hydroxyapatite/polyurethane composite	Levofloxacin	–	–	*In vivo*	*S. aureus*	CFU count	Rabbits	After 12 w	–	–	–	[149]
Mesoporous silica microspheres/nano-hydroxyapatite/polyurethane	Levofloxacin	–	–	*In vitro*	*S. aureus, E. coli*	Agar diffusion, CFU count	–	–	Pre-osteoblastic MC3T3-E1	Increased	Increased after 14 d	[149]
3D printed composite scaffolds based on PVA/Gold nanoparticles (AuNP)/	Ampicillin	–	–	*In vitro*	*S. aureus*	Agar diffusion	–	–	MC3T3-E1 mouse osteoblast cells	Increased	Increased at 21 d	[154]
Polyurethane (PU) scaffold loaded with nano-hydroxyapatite (HA/PU-CF)	Ciprofloxacin	–	Sustained/14 d	*In vitro*	*S. aureus, E. coli*	Agar diffusion, MIC	–	–	BMSCs	Increased	–	[155]
Nano-hydroxyapatite pellets	Vancomycin	–	Sustained/25 d	*In vivo,* *In vitro*	*S. aureus,* *E. coli,* MRSA	Agar diffusion	Rabbits	3 months	–	–	–	[189]
Hydroxyapatite nanoparticles	Ciprofloxacin (5 mg/mL)		aSustained/60 d	*In vitro*	*S. aureus, E. coli*	Agar diffusion	–	–	MG-63 cells	–	–	[162]
Hydroxyapatite nanoparticles	Ciprofloxacin (10 mg/mL)		aSustained/60 d									
Hydroxyapatite nanoparticles	Ciprofloxacin (20 mg/mL)		aSustained/60 d									
Calcium phosphate cements loaded with human lactoferrin 1-11	Gentamicin	–	–	*In vivo*	*S. aureus*	CFU count	Rabbits	After 3 w	–	–	–	[165]
Calcium phosphate beads	Tobramycin	30 mg/mL	10.0 μg/mL/168 h, sustained 8 w	*In vivo*	*S. aureus*	CFU count	Rabbits	After 28 d	–	–	–	[166]
3D-printed calcium phosphate scaffold CaPS coated with poly(lactic co-glycolic) acid	Rifampin, Sitafloxacin	–	Rifampin = 70 μg/mL/48h, Sitafloxacin = 55 μg/mL/48h, Sustained/2 w	*In vivo*	*S. aureus*	Agar diffusion	Mice	After 3 and 10 w	–	–	–	[167]
Bioactive glasses												
Gelatin-coated meso-macroporous	Levofloxacin, Vancomycin, Rifampicin, Gentamicin	–	a	*In vitro*	*S. aureus, E. coli*	Agar diffusion	–	–	–	–	–	[175]
Bioactive glass nanoparticles	Gentamicin, Ampicillin	Gentamicin = 33 μg/mL, Ampicillin = 30 μg/mL	–	*In vitro*	*S. aureus, E. coli*	Agar diffusion	–	–	Osteoblast	Increased	–	[176]
Bioactive borate glass VCS	Vancomycin	80 mg/g	68.36–74.18 %/48h, sustained/16 d	*In vivo,* *In vitro*	MRSA	MIC	Rabbit	After 8 w	–	–	–	[177]
Bioactive borate glass VBG	Vancomycin		32.6–35.9 %/48h, sustained/18 d	*In vivo,* *In vitro*	MRSA	MIC	Rabbit	After 8 w	–	–	–	

**Abbreviations:** ALP: alkaline phosphatase, CaP: calcium phosphate, CFU: colony forming unit, EE: entrapment efficiency, MIC: minimum inhibitory concentration, MRSA: Methicillin-Resistant *Staphylococcus aureus*, PVA: polyvinyl alcohol.

a The exactvalues are not reported numerically in the text of referenced article and drug release patterns are presented in the graph.

##### 4.1.2.4 Poly ε-caprolactone (PCL)

Poly ε-caprolactone (PCL) is a biocompatible and biodegradable polymer member of aliphatic polyester, which is widely studied for biomaterial application [141,142]. PCL shows a low melting point (60 °C), which is beneficial for 3D printing [143]. In addition, PCL showed favorable mechanical features with high-level elasticity and great extension at the break, which is attractive for developing biomaterials and composites for bone tissue engineering and as delivery systems for different drugs [144]. Low hydrophilicity, poor mechanical features, low degradability, and insufficient osteoconductive activity are addressed as some limitations of PCL scaffolds. One approach to improve undesirable properties of PCL is to design nanoscaffolds reinforced with biocompatible compounds [145]. PCL has been used to develop vancomycin–impregnated membranes to control bone infection caused by *S. aureus* effectively, and in the regeneration of bone in a critical bone defect animal model [146]. The 3D printing PCL/PLGA has been used as the delivery system of tobramycin. 3D printing PCL/PLGA/tobramycin has shown *in vitro* inhibitory effects on different pathogens and reduced the transcription levels of proinflammatory mediators of TNF-α and IL-6 in the RAW 264.7 cells line. The transcription levels of these cytokines have been reduced with the increase of loading levels of tobramycin on the scaffolds. This indicates the inflammatory response of RAW 264.7 cells were decreased by the released tobramycin. The 3D printed PCL/PLGA/tobramycin scaffold significantly decreased the edema and inflammation caused by *S. aureus* infection in a rat model with femur chronic osteomyelitis and induced new bone generation eight weeks after the implantation [147]. PCL-silica sol-gel hybrid material has been fabricated as the system for the encapsulation and delivery of rifampicin. Rifampicin has been successfully loaded within the PCL-silica sol-gel hybrid without detectable degradation. It has shown significant *in vitro* inhibitory effects on the most common bacterial agents of bone infections. The fast release of silicates from PCL-silica sol-gel hybrid has been described to be related to *in vitro* induction of cell proliferation and osteoblastic differentiation. Concurrently, rifampicin has been delivered for several weeks with significantly inhibiting tested bacteria [148].

#### 4.1.3 Other polymers

Composite scaffolds of levofloxacin-loaded MSNs microspheres/nano-hydroxyapatite/polyurethane have shown a significant effect on treating chronic osteomyelitis caused by *S. aureus.* Levofloxacin released from the MSNs microspheres/nano-hydroxyapatite/polyurethane scaffolds has inhibited the development of chronic osteomyelitis shortly after embedding. After one week and three weeks from implantation, the significant inducing new bone formation has been reported by levofloxacin-loaded MSNs microspheres/nano-hydroxyapatite/polyurethane [149]. Levofloxacin-loaded MSNs microspheres/nano-hydroxyapatite/polyurethane composite scaffold has been shown to have promising *in vitro* biocompatibility, triggered MSCs osteogenic differentiation, induced proliferation and differentiation of MC3T3-E1, and suppressed apoptosis [150]. Therefore, the dual functions of anti-microbial and increased osteogenesis of levofloxacin-loaded MSNs microspheres/nano-hydroxyapatite/polyurethane composite scaffold have made it attractive to future clinical investigation.

Wu prepared a spatiotemporal drug-releasing polydopamine-functionalized core/shell MSNs drug delivery system for loading silver nanoparticles and dexamethasone to fabricate and introduce into poly-L-lactic acid (PLLA) scaffolds for the treatment of osteomyelitis. The scaffold showed excellent antibacterial performance against *S. aureus* and *E. coli*, and also increased the bone differentiation of stem cells [151].

Gold nanoparticles give an attractive compound for investigation due to high stability, low toxicity, and uncomplicated to produce nanoparticles and display several attractive properties such as assembly of numerous forms and significant size effect [152]. Gold nanoparticles have been shown to improve the characteristics of scaffold composites and significantly induce osteogenic activities. Gold nanoparticles also may affect some metabolic pathways of the human body, which directly or indirectly induce bone tissue regeneration [153]. Composite scaffolds based on f polyvinyl alcohol/gold nanoparticles/ampicillin have been developed with antimicrobial effects on *S. aureus*. Polyvinyl alcohol/gold nanoparticles, polyvinyl alcohol/ampicillin, and polyvinyl alcohol/gold nanoparticles/ampicillin have shown higher viability than polyvinyl alcohol. Inhibition of bacterial growth has been reported for scaffolds containing ampicillin and gold nanoparticles/ampicillin. Polyvinyl alcohol/ampicillin-containing scaffolds have exhibited a higher *in vitro* antimicrobial effect than polyvinyl alcohol/gold nanoparticles/ampicillin. Furthermore, MC3T3-E1 cell viability has not been affected by polyvinyl alcohol/ampicillin and polyvinyl alcohol/gold nanoparticles/ampicillin and exhibited significantly higher cell viability than the polyvinyl alcohol at days 7, 14 and 21, respectively. Osteoblastic differentiation and calcium depositions have also been induced by composite scaffolds based on polyvinyl alcohol/gold nanoparticles/ampicillin. The stimulation of the ERK/MAPK cell signaling pathway by gold nanoparticles has been described as an essential mechanism for inducing this increased osteogenic response [154].

A multi-functional scaffold composed of polyurethane (PU) scaffold loaded with nano-HAP and ciprofloxacin has exhibited appropriately controlled drug release patterns after 14 days and considerable continuous antibacterial properties against *S. aureus* and *E. coli*. The addition of HAP into PU scaffolds has been reported to increase *in vitro* osteoblastic differentiation of the BMSCs [155].

### 4.2 Inorganic scaffolds

#### 4.2.1 Ceramic scaffolds

Ceramics are inorganic, non-metallic, and crystalline compounds, which can be classified according to their interaction with biological molecules as bioinert and bioactive. In contrast to bioinert ceramics, bioactive ceramics are characterized by the ability to induce adherence to living cells and tissue. The most common bioactive ceramics in bone tissue biomaterial, also referred to as bioceramics, hydroxyapatite (HAP), bioactive glass, and β-tricalcium phosphate because of calcium salts that promote the formation and precipitation of calcium phosphates in bone tissue [156,157]. Ceramics have been widely studied as implant materials because of their potential to be prepared into various shapes, along with their high compressive strength, variable porosity, and activity in the host body [158].

#### 4.2.2 Hydroxyapatite (HAP)

HAP is a member of the apatite family (composed of calcium and phosphates) with a chemical formula Ca10(PO4)6(OH)2. HAP is prepared from both biological and synthetic origins [159]. HAP is thermodynamically resistant in its crystalline form in the human body liquids and shows a highly comparable conformation to bone minerals. HAP can interact with bone cells without prompting any local toxicity or general side effects and immunity response. For these reasons, HAP has been extensively studied for application in bone tissue engineering principally in orthopedic, odontology, and as the coating material for metallic implants [160]. Multi-functional scaffold composed of polyurethane (PU) scaffold loaded with nano-HAP and ciprofloxacin has been exhibited appropriate controlled drug release patterns after 14 days and considerable continuous antibacterial properties against *S. aureus* and *E. coli*. The addition of HAP into PU scaffolds has been reported to increase *in vitro* osteoblastic differentiation of the BMSCs [155]. The results of the *in vivo* study indicated an effective and safe controlled-release pattern of vancomycin from nano-HAP pellets. Vancomycin-loaded HAP pellets have been shown to exhibit the ability to repair bone defects and control chronic osteomyelitis induced by MRSA in rabbit models [161]. Ciprofloxacin-loaded HAP nanoparticles with continued and lengthy release patterns of ciprofloxacin have inhibited *S. aureus* and *E. coli*. It was reported that the concentration of ciprofloxacin loaded on HAP can be easily controlled by altering the primary concentration during the preparation. The increasing loaded amount enhanced the sustained and prolonged release of ciprofloxacin. The in situ loading of ciprofloxacin onto HAP nanoparticles has not shown significantly effects on the bioactivity and cytocompatibility of HAP, whereas it increased antibacterial effects against osteomyelitis. Therefore, ciprofloxacin loaded HAP nanoparticles can be promising option for the osteomyelitis treatment [162].

#### 4.2.3 Calcium phosphates

Calcium phosphate is the main ingredient of bone tissue and teeth and plays a vital role in the human body [163]. The interest in calcium phosphate-based biomaterials usage in bone tissue engineering is due to a high-level similarity with composition to the bone mineral (a calcium phosphate in the form of carbonate apatite), and considerable biodegradability, biocompatibility, bioactivity, and osteogenic activity [164]. Biodegradable calcium phosphate cements are widely studied as carriers for the controlled delivery of various antimicrobial agents. Calcium phosphate cement with human lactoferrin 1-11 (hLF1-11), an antimicrobial peptide, or gentamicin has been developed to kill *S. aureus.* The hLF1-11, and gentamicin reduced the bacterial count number in infected femora of infected animal model [165]. It has been fabricated tobramycin-loaded calcium phosphate beads that potentially inhibited the growth of *S. aureus*. Tobramycin (30 mg/mL) has been incorporated into calcium phosphate beads by dipping technique, and the effectiveness of tobramycin-loaded calcium phosphate beads has been detected in an animal model of osteomyelitis that exhibited significant inhibitory effects on the growth of *S. aureus* in infected bone tissue than the controls [166]. *S. aureus* causing osteomyelitis has been treated with 3D-printed calcium phosphate loaded with rifampin and sitafloxacin. Calcium phosphate was prepared with either sitafloxacin, rifampin, or a combination of sitafloxacin-sitafloxacin/scaffold and rifampin/scaffold. To attain continuous drug release from calcium phosphate, scaffolds were coated with two layers of drug-containing poly D, L-lactide-co-glycolide [167].

#### 4.2.4 Bioactive glasses

Bioactive glasses are the most significant bioceramics for biomedical usage and have attracted considerable attention due to incompatibility, and safety, and are chemically stable in biological environments [168,169]. Bioactive glasses have shown antimicrobial effect and since it increases the pH and osmolarity locally, thus generating an unfavorable condition for microbial growth [170]. Bioactive glasses have also exhibited both osteoconductive and autoinduction effects and are widely studied for a variety of biomaterial applications and bone tissue engineering. Nevertheless, despite their exceptional bioactive properties, the main drawbacks of bioactive glasses are their low fracture toughness and low mechanical strength [171].

After placing bioglass at the damaged site of a host body, its glass surface is hydrated by body fluids, thereby starting a conversion response, and a thin layer of HA is shaped on the glass surface that condenses over time, triggering mineralization of the matrix [172]. Bioactive glass exhibits an inhibitory effect on bacteria without inducing microbial resistance, together with a considerable inhibitory effect against biofilm [173].

The antibacterial activity of bioactive glass S53P4 has been demonstrated in treating chronic osteomyelitis of the long bones caused by MRSA and *S. epidermidis*, *P. aeruginosa,* and *A. baumannii* [174]. It has been fabricated the scaffolds based on mesoporous bioactive glasses before and after the addition of 4 % ZnO and loaded with saturated and minimal inhibitory concentrations of vancomycin, levofloxacin, gentamicin, or rifampicin on inhibiting *S. aureus* and *E. coli,* and treat bone infection [175]. Dual antibiotics gentamicin sulfate and sodium ampicillin on the bioglass nanoassembly sample have been developed to effectively inhibit the growth of *E. coli* and *S. aureus* causing osteomyelitis [176]. Bioactive borate glasses have shown effective delivery systems for vancomycin to eradicate osteomyelitis [177].

#### 4.2.5 Demineralized bone matrix (DBM)

One of the major therapeutic options for bone grafting is the demineralized bone matrix (DBM), which exhibits osteoinductive and osteoconductive properties for the regeneration and engineering of bone tissue [178,179]. Given that the most common clinical DBM formats are moldable paste or putty, various carriers have been proposed to be loaded into DBM to ensure local drug delivery after implantation [180].

To combine the effects of bone repair and prevention/eradication of infection, Govoni et al. prepared formulation of DBM paste for vancomycin site-specific controlled delivery ensuring a therapeutically active amount. Then, pure vancomycin or vancomycin-PLGA-HA micro/nano-carrier was loaded into DBM paste to monitor the drug release and the antimicrobial effect of the prepared scaffold against *S. aureus*. This study showed the potential of the DBM-carrier-antibiotic system in inhibiting the growth of bacteria with precise antibiotic release [181].

Retinam et al. developed an electrospun nanobiomembrane (ENBM) from nano DBM, polyvinyl alcohol and added carbon nanoparticles (to create more strength) by electrospinning. This study proved the bone formation by ENBM in the regeneration of bone tissue [182].

In another study, nanoceramic composite (NCC) was designed using the DBM, collagen, olive leaves extract, and curcumin nanoparticles. The study evaluated using NCC as a functionalized implant using natural resources in tissue engineering [183].

## 5 Future perspective

An effective local co-delivery system should exhibit a sustained and prolonged release pattern of incorporated antimicrobial agents and osteoinductive compounds to overcome infection-related damages and tissue regeneration. In addition, the potential interference between the antimicrobial agents and osteoinductive factors, either during the fabricate or after delivery to host tissue need to be evaluated. The appropriate co-delivery system with dual therapeutic effects should exhibit other properties, including biocompatibility, biodegradability, desirable mechanical characteristics, and stable incorporation with host bone tissue. One of the most determining factors in the efficiency of a drug delivery system in antibiotic therapy of osteomyelitis is usage an appropriate antibiotic. In various studies, drug delivery systems have been designed for different antibiotics, the most common of which are vancomycin, ciprofloxacin, gentamicin, clindamycin, and rifampin. The effectiveness of these antibiotics is principally related to the pathogens causing the infection. MRSA is the most common causative pathogenic in cases of osteomyelitis. MRSA strains show a high level of resistance to β-lactams and other classes of antibiotics, such as aminoglycosides, macrolides, and fluoroquinolones. Therefore, some drugs, such as ciprofloxacin, clindamycin and aminoglycosides may not have satisfactory treatment outcomes in MRSA-caused cases. The drug of choice for infections caused by these strains is vancomycin and some new antibiotics, such as linezolid. Another potential option for treating infections caused by MRSA is using a combination of antibiotics to achieve synergistic effects. Therefore, drug delivery systems for the simultaneous delivery of antimicrobial compounds can be help treat bone infections by MRSA. Since the determining role of microbial biofilms as well as the intracellular forms of pathogen that cause drug-resistant osteomyelitis, the selection of effective antibiotics on these forms and the investigation of antimicrobial effects and the ability to penetrate the cell and inhibit intracellular forms must be considered in the design and delivery systems of drugs.

## 6 Conclusion

Current advances in biomedicine and tissue engineering have contributed to developing potential therapeutic approaches for osteomyelitis management using nano-scaffolds. To prepare the scaffolds used in the drug delivery for osteomyelitis, various natural and synthetic compounds have been studied. Despite being biocompatible and non-toxic, natural polymers may not show ideal mechanical properties. This can be improved by preparing composites from a combination of natural polymers, and synthetic materials with high strength. Various nano-scaffolds have been prepared using natural polymers such as silk, collagen, gelatin, fibrinogen, chitosan, cellulose, hyaluronic, alginate, and synthetic compounds such as PLA, PGA, PLGA, and PCL. In addition to incorporated antimicrobial agents and the content of scaffolds, the physical and chemical characteristics of the prepared delivery systems are a determining factor in their effectiveness in treating osteomyelitis. To date, antimicrobial effects and osteoinductive properties of various biomaterials have been reported. However, few cases displayed both activities in the same compound, other than, until applied as a co-delivery approach.

## CRediT authorship contribution statement

**Mina Yekani:** Writing – review & editing, Writing – original draft, Software, Methodology, Investigation. **Solmaz Maleki Dizaj:** Writing – review & editing, Writing – original draft, Investigation. **Simin Sharifi:** Writing – review & editing, Writing – original draft, Software. **Hossein Sedaghat:** Writing – review & editing, Writing – original draft. **Mahmood Saffari:** Writing – review & editing, Validation, Project administration. **Mohammad Yousef Memar:** Writing – review & editing, Writing – original draft, Supervision, Investigation, Conceptualization.

## Disclosure statement

The authors reported no potential conflict of interest.

## Data availability statement

Not applicable.

## Declaration of competing interest

The authors declare that they have no known competing financial interests or personal relationships that could have appeared to influence the work reported in this paper.
